# M. Velpeau and M. Jules Guérin

**Published:** 1867-01

**Authors:** 


*M. Velpeau and M. Jules Guérin.*—Both of these surgeons are members of the Academy of Medicine of Paris; the former, however, as every one knows, is professor at the Faculty, whilst the latter is unconnected with official teaching. For the last thirty years a somewhat bitter warfare has occasionally been carried on between these two eminent men, and one of the fiercest disputes is going on at the present time before the Academy.

M. Jules Guérin maintains, both in his speeches and in the *Gazette Medicale de Paris*, of which he is the able editor, that he is the *inventor* of subcutaneous surgery; and M. Velpeau, though allowing his opponent some share in the merit of having extensively applied subcutaneous sections, refuses, year after year, to admit the claim. It so happens that both belligerents are gifted with great elocutionary powers, that both are extensively read, and that both are wonderfully persevering and unyielding.

Those interested in subcutaneous surgery will find a mass of valuable information in the speeches recently delivered; and will, perhaps, remain convinced that, in the face of Stromeyer's and Schönbein's labors, the claim of discovery can hardly be defended. They will at the same time allow that great credit is due to M. Guérin for his valuable orthopædic labors. Not the least of the results obtained by the latter surgeon is the healing of large wounds by first intention, through pneumatic occlusion.

At the bottom of the debate lies the fact that M. Velpeau represents the scholastic interest, the pride of the faculty, and the professional dignity, against the really talented doings of an independent surgeon, unconnected with official position. The late M. Amussat, whose name is well known in the surgical world, succeeded, like M. Guérin, in becoming an eminent operator without attaining hospital or university distinction. These cases, however, are rare; for it requires an unusual amount of skill, perseverence and surgical tact to triumph over the difficulties besetting private practice. M. Guérin is a specialist; he has done good service in his orthopædic establishment. Like Behrend, of Berlin, too, he is not slow in publishing his success, and is ever ready to fight hard battles in order to uphold his well earned reputation.—*Ibid.*

